# Ablation of *Gabra5* Influences Corticosterone Levels and Anxiety-like Behavior in Mice

**DOI:** 10.3390/genes14020285

**Published:** 2023-01-21

**Authors:** Linn Amanda Syding, Agnieszka Kubik-Zahorodna, David Pajuelo Reguera, Petr Nickl, Bohdana Hruskova, Michaela Kralikova, Jana Kopkanova, Vendula Novosadova, Petr Kasparek, Jan Prochazka, Jan Rozman, Rostislav Turecek, Radislav Sedlacek

**Affiliations:** 1 Laboratory of Transgenic Models of Diseases, Institute of Molecular Genetics of the CAS, 25250 Vestec, Czech Republic; 2 Czech Centre for Phenogenomics, Institute of Molecular Genetics of the CAS, 25250 Vestec, Czech Republic; 3 Department of Auditory Neuroscience, Institute of Experimental Medicine of the Czech Academy of Sciences, 14220 Prague, Czech Republic

**Keywords:** corticosterone, GABA receptor, anxiety, behavior, mouse model

## Abstract

Stress responses are activated by the hypothalamic-pituitary-adrenal axis (HPA axis), culminating in the release of glucocorticoids. During prolonged periods of secretion of glucocorticoids or inappropriate behavioral responses to a stressor, pathologic conditions may occur. Increased glucocorticoid concentration is linked to generalized anxiety, and there are knowledge gaps regarding its regulation. It is known that the HPA axis is under GABAergic control, but the contribution of the individual subunits of the GABA receptor is largely unknown. In this study, we investigated the relationship between the α5 subunit and corticosterone levels in a new mouse model deficient for *Gabra5*, which is known to be linked to anxiety disorders in humans and phenologs observed in mice. We observed decreased rearing behavior, suggesting lower anxiety in the *Gabra5^−/−^* animals; however, such a phenotype was absent in the open field and elevated plus maze tests. In addition to decreased rearing behavior, we also found decreased levels of fecal corticosterone metabolites in *Gabra5^−/−^* mice indicating a lowered stress response. Moreover, based on the electrophysiological recordings where we observed a hyperpolarized state of hippocampal neurons, we hypothesize that the constitutive ablation of the *Gabra5* gene leads to functional compensation with other channels or GABA receptor subunits in this model.

## 1. Introduction

Stress is defined as a state of mental or physical strain resulting from demanding circumstances mirroring internal or external conditions threatening the homeostasis of the organism [1,2]. The hypothalamic-pituitary-adrenal-axis (HPA-axis) is the main system mediating stress responses through physiological or behavioral adaptations. The response to stress starts with the stimulation of hypophysiotropic neurons, innervated by afferents from limbic brain regions in the paraventricular nucleus (PVN) of the hypothalamus [3]. The HPA-mediated response culminates in the release of glucocorticoids through several intermediate steps [4] that bind to their ubiquitously expressed receptors, activating the transcription of target genes [5].

The relationship between glucocorticoids, commonly referred to as cortisol in humans and corticosterone in rodents, and increased anxiety and behavioral impairments has been established [6]. Increased secretion of glucocorticoids is tied to increased general anxiety, depressive disorders, and social anxiety [7]. Although the physiology behind the stress response and its role in psychological disorders has been extensively studied, there are still gaps in the knowledge of the contribution of the GABA(A) subunit in terms of its regulation.

The PVN receives considerable GABAergic innervation from local hypothalamic regions and areas of the amygdala that exert a substantial inhibitory tone on the HPA axis [8]. Limbic regions such as the hippocampus and prefrontal cortex do not directly innervate the PVN but control HPA action via projections to GABAergic nuclei surrounding the PVN [9]. The functional role of GABA in regards to corticosterone secretion was shown when microinjection of the GABA agonist muscimol into the hypothalamus resulted in inhibited secretion of glucocorticoids [10]. The GABA(A) receptor is a ligand-gated chloride ion channel that, when activated by GABA or a positive allosteric modulator leads to hyperpolarization of the cell, thus being an inhibitory neurotransmitter receptor in the CNS [11]. The functionality of the heteropentameric GABA(A) receptor, including the pharmacological sensitivity, subcellular location, and channel properties, varies according to subunit assembly [12]. The GABA(A)R subunit composition in terms of regulating the stress response still has left to be elucidated as the contribution of single subunits is still being discovered. Interestingly, it was shown that in stressed mice, GABA has an excitatory effect on the HPA axis potentiated via the GABA(A) delta subunit-containing receptors [13]. The individual contribution of other subunits with regard to the stress response is still unclear.

Perturbations in the *GABRA5* gene, encoding the α5 subunit, were found in patients with panic disorder, and heterozygous expression of the gene is commonly present in patients with Angelman syndrome, where anxiety and autism are commonly reported conditions [14,15]. Assumptions about the function of the gene were corroborated by corresponding phenotypes in *Gabra5^−/−^* mutant mice and α5 pharmacological inhibition [16,17,18]. In addition, high α5 subunit expression is described in the hippocampus, part of the limbic system, which is rich in glucocorticoid receptors [19,20].

Besides the hippocampus, the receptors are found in the cortex, hypothalamus, and amygdala [21], although at lower levels. The brain regions with substantial α5 expression are all involved in the fear circuitry, which is a central component of anxiety [22]. As outlined above, the hypothalamus is the master regulator of the HPA axis, where the cascade starts [23]. However, structures such as the hippocampus play an important role when anxiety-like behavior is exhibited [24,25]. It has been shown that α5 GABA(A) receptors generate tonic inhibitory conductance in CA1 hippocampal pyramidal neurons [26]. Taken together, these findings make the *Gabra5* gene an interesting candidate for studies on glucocorticoids and anxiety-like behavior.

Based on the effects of conditional *Gabra5^−/−^* models and pharmacological α5 GABAAR inhibition on anxiety-like behavior and the link between glucocorticoids and anxiety, we aim to elucidate the putative relationship between the α5 subunit and corticosterone in a new mouse model based on targeting critical exon 3 to functionally ablate the gene. Our model is genetically similar to the one generated by Collinson et al. (2002) [27], but was produced as part of the efforts of the International Mouse Phenotyping Consortium (IMPC, www.mousephenotype.org) which aims to generate and phenotype a knockout mouse model for every protein-coding gene on a harmonized genetic background and adopts shared standard operating procedures for phenotyping.

We assessed whether housing conditions for single- and group-housed individuals affected corticosterone levels. We also assessed general anxiety using open field and elevated plus maze tests, while locomotor activity and rearing behavior under home cage conditions, and energy expenditure was evaluated by indirect calorimetric devices. Hippocampal-dependent and -independent memory and learning were assessed with contextual and delayed cued fear conditioning, respectively. Finally, we employed electrophysiological recordings for functional evaluation of the mouse model.

To summarize, we hypothesized that an ablation of the α5 subunit would result in increased corticosterone secretion and increased anxiety-like behavior due to the overall increased excitability of the GABAergic neurons. Altogether, we observed decreased corticosterone levels in *Gabra5^−/−^* mice and decreased rearing behavior, suggesting lower anxiety levels as well as hyperpolarization of pyramidal hippocampal neurons in α5 deletion neurons. Based on the results obtained, we believe functional compensation may be present in this model.

## 2. Materials and Methods

### 2.1. Mouse Husbandry

All animal models and experiments used in this study were ethically reviewed and performed following European Directive 2010/63/EU and were approved by the Czech Central Commission for Animal Welfare. Mice were housed in individually ventilated cages (Techniplast, Buguggiate, Italy) in a barrier facility. Mice were genotyped when they were 14–21 days old. All animals were kept at 22 ± 2 °C with a 12 h dark and light cycle and were tested during the light period, provided with mouse chow (Altromin 1314, Altromin, Lage, Germany) and water ad libitum. During housing, the mice were group-housed with two to six animals of the same sex and genotype per cage.

### 2.2. Model Generation

The *Gabra5* knockout mouse on C57BL/6N background used for this study was generated by targeting exon 3 of the *Gabra5* gene (transcript Gabra5-201 ENSMUST00000068456.8) for an exon deletion by using the CRISPR/Cas9 technique at the Institute of Molecular Genetics, Prague. The gRNAs were generated using (http://crispor.tefor.net/, accessed on 25 June 2018) where the gRNAs with the highest score were selected. The gRNAs for electroporation were the following: Gabra5 forward 5′- GGCCGCAGTCTGTTGTCATA-3′ and Gabra5 reverse 5′- ACTAGTTCTGTACAAGACGA-3′. The gRNAs were introduced to the fertilized oocytes of the C57BL/6N strain and transferred into pseudo-pregnant foster mice. Putative founders were analyzed by PCR, gel electrophoresis, and sequencing. One animal harboring a 903 nucleotide deletion spanning exon 3 and parts of introns 2 and 3 was chosen for subsequent breeding to F1 progeny. Genotyping was performed using the following primers: forward: 5′- TACAGAAGCAAGGGGTTCAGG -3′, reverse: 5′- GCCTCCCTGTTCTTATTGTCG-3′ with Ta 65 °C.

### 2.3. Western Blotting

Hippocampi were dissected and flash-frozen in liquid nitrogen. The tissue was lysed in RIPA buffer (0.05 M Tris-HCl, pH 8, 0.15 M NaCl, 0.5% deoxycholic acid, 1% NP-40, and 0.1% sodium dodecyl sulfate (SDS)), cOmplete™, EDTA-free Protease Inhibitor Cocktail (Roche, 5056489001), and PhosSTOP™, phosphatase inhibitor tablets (Roche, 4906845001). Lysates were sonicated and cleared by centrifugation. Protein concentration was determined using the Pierce™ BCA Protein Assay Kit (ThermoScientific, 23225). 8μg of protein from the hippocampus was denatured in a reducing sample buffer, separated by SDS-PAGE (polyacrylamide gel electrophoresis) gels, and blotted to nitrocellulose membranes (Bio-Rad). The primary antibodies used were rabbit anti-Gabra5 (1:4000, Synaptic Systems, 224 503) and mouse anti-Vinculin (1:10,000, Invitrogen, 700062). Blots were washed with PBS-T, and detection was performed with SuperSignal™ West Pico PLUS Chemiluminescent Substrate (ThermoScientific, 34579).

### 2.4. Fecal Corticosterone Assessment and Housing

For the detection of fecal corticosterone metabolites and the effect of housing, we used a total of six animals at 11–12 weeks, one per sex, genotype, and housing-condition. The animals were placed in single-housing for those selected, and group animals were kept in their home cage at six individuals each. Egested feces were collected at 8 am at 24 h and 48 h after separation. The fecal samples were dried overnight at 60 °C and weighed; 1 ml of 80% methanol was added per 50 mg of feces. The samples were seal homogenized and then left shaking at 1000 rpm at room temperature overnight. The fecal samples were spun down at 4 °C at 2500 rpm for 10 min and the supernatant was moved to a new tube. The fecal corticosterone metabolites were assessed using corticosterone ELISA (Biovendor, RTC002R) according to the manufacturer’s protocol.

### 2.5. Testosterone Sampling and Elisa

Males at 11 w of age were used for testosterone analysis. The animals were either group-housed at six animals per cage or single-housed. After 24 h and 48 h of separation at 10 am, we collected 25 μL of blood from the tail in lithium/heparin microvettes (Sarstedt, 16.443) and the blood was spun down at 2500 g for 10 min at 4 °C to obtain plasma. We analyzed the testosterone levels using testosterone ELISA measurements according to the manufacturer’s protocol (Biovendor, RTC001R).

### 2.6. Open Field

The activity of the animals in a novel environment and the level of anxiety displayed were evaluated in open field tests as previously described [28]. The area of the open field was a square of 42 × 42 cm uniformly illuminated with a light intensity of 200 lux in the center of the field. The testing arena was virtually divided into periphery and center zones, where the center zone constituted 38% of the whole arena. Each mouse was placed in the corner of the arena for a 10 min period of free maze exploration. The time spent in each zone, the distance travelled, and other indices were automatically computed based on video recordings (Viewer software 3.0.1.452, Biobserve GmbH, Germany). In total, 23 WT mice and 21 *Gabra5^−/−^* were tested with an approximate 1:1 gender ratio at 10–11 w. Additionally, a number of supported and unsupported rearings were manually assessed based on video recordings.

### 2.7. Elevated Plus Maze

The elevated plus maze (EPM) apparatus consisted of two closed and two open elevated arms, with a light intensity of 60 lux in the center of the maze. The animals were placed in the center and were left to explore the EPM for five minutes. The total time spent in the open and closed arms and the center of EPM were tracked and evaluated automatically (Viewer software 3.0.1.452, Biobserve GmbH). Additionally, head-dipping and rearings were counted manually based on video recordings. We tested 11 animals per experimental group at 12 w.

### 2.8. Fear Conditioning

To evaluate the mice’s ability to learn and remember associated environmental cues and aversive experiences, we employed the contextual and delayed cued fear conditioning test [29]. The mice were placed in a chamber with white light (40 lux) for 4 min when the baseline freezing time was monitored. Following mice were presented with pairing of the conditioned stimulus (CS, 20 s of 4 kHz pure tone at 77 dB) and unconditioned stimulus (CS, 1 s long electric shock of 0.6 mA constant current, Ugo Basile, Gemonio, Italy). The shock came with the termination of the CS. Animals were tested for contextual memory 24 h after training. They were reintroduced to the training context, and the behavior was recorded for 6 min with no CS or US presentation. The dependent measure of freezing time was automatically estimated by software. Delayed cue memory was tested four hours later in a novel context with a different cage wall pattern, smooth floor texture, and menthol scent. The freezing response to the CS was monitored for 2 min. All animals were between 9–10 w at the time of testing.

### 2.9. Slice Preparation

For electrophysiology experiments, transversal hippocampal slices were prepared from P36–P44 WT and *Gabra5*^−/−^ mice. Neurons were pooled in an equal gender ratio for both experimental groups. Animals were decapitated in accordance with the Animal Protection Law of the Czech Republic (compatible with European Community Council Directives 86/609/EEC). The brains were excised in ice-cold low Ca^2+^ artificial CSF (aCSF) containing the following (in mM): 130 NaCl, 3.5 KCl, 3 MgCl_2_, 0.5 CaCl_2_, 10 glucose, 1.25 NaH_2_PO_4_, 24 NaHCO_2_, 0.5 ascorbic acid, 3 myo-inositol, and 2 sodium pyruvate; bubbled with 5% CO_2_/95% O_2_ to pH 7.3. Slices (300 μm thick) were cut in the low Ca^2+^ aCSF using a VT1200S vibratome (Leica). After sectioning, the connection between CA1 and CA3 regions was cut; the slices were incubated at 34 °C for 60 min and then stored at room temperature (21–23 °C) in a submerged chamber with aCSF.

### 2.10. Electrophysiology

During recording, slices were perfused with a standard aCSF containing (in mM): 125 NaCl, 2.5 KCl, 1 MgCl_2_, 2 CaCl_2_, 25 glucose, 1.25 NaH_2_PO_4_, 25 NaHCO_2_; bubbled with 5% CO_2_/95% O_2_ to pH 7.3. The recordings were performed at physiological temperature (35°C). Whole-cell patch-clamp recordings were performed in the stratum pyramidale of CA1; during recording, neurons were viewed using infrared and differential interference contrast optics. Borosilicate glass electrodes (~2–3 MΩ) were filled with a solution containing the following (in mM): 107 K-gluconate, 32.5 KCl, 5 EGTA, 10 HEPES, 4 MgATP, 10 Tris-phosphocreatine, 0.6 NaGTP, pH 7.25, 295 mOsm). The aCSF was supplemented with 5 μM (±)-CPP, 10 μM DNQX, 1 μM CGP 54,626 and 5 μM SR-95531 to block ionotropic glutamate, GABA(B) and synaptic GABA(A) receptors. 10 nM L-655,708 was used to inhibit the extrasynaptic α5-containing GABA(A) receptors [30]. 5μM GABA was used to replace the residual GABA concentration in the hippocampus [31]. Drugs were stored in frozen aliquots, and solutions were freshly prepared on the day of the experiment.

Membrane potentials were recorded with an Axopatch 200B amplifier (Molecular Devices); signals were filtered at 10 kHz, digitized at 50 kHz, and acquired using pCLAMP 11 software (Molecular Devices). Voltages are corrected for junction potentials of 12 mV. Membrane resistance was derived from the amplitude of neuronal voltage responses to −10 pA current step injections. Current steps of increasing amplitude (1 s, 0.05–0.215 nA) were injected into neurons to determine the rheobase of their action potentials. Data were analyzed using pCLAMP 11 and GraphPad Prism 9 software.

### 2.11. Indirect Calorimetry

Indirect calorimetry was performed using an 8-cage multiplex setup, including the monitoring of food and water uptake and physical activity (distance travelled and rearing, PhenoMaster, TSE Systems, Bad Homburg, Germany, software version v.7.1.2). Before starting the indirect calorimetry measurements, we performed a complete calibration protocol for the gas analyzers according to the manufacturer’s recommendations using compressed air, with CO_2_ at 1% and N_2_ at 100%. We weighed the mice before introducing them into the calorimetric cages. Mice were measured individually. Mice had ad libitum access to water and food, (Altromin 1314, Lage, Germany). The volume of bedding material was limited to approximately 150 mL per cage during indirect calorimetry measurements to properly detect locomotor activity of the mice by infrared beam breaks frame surrounding the cage in the horizontal plane (ActiMot2).

The mice were individually housed in a multiplex system with eight cages plus a reference cage. In total, 8 animals per sex and genotype at an age of 12 w. Sampling frequency to measure the CO_2_ and O_2_ gas measurements was every 15 min. The environmental conditions inside the climatic chamber were 23 degrees centigrade, 55% relative humidity, and a light cycle of 12 h of light and 12 h of darkness synchronized with the animal facility where the mice were housed. The following 72 h period was used for measurements of the CO_2_ production and O_2_ consumption, where the energy expenditure (EE), locomotor activity, including rearing behavior, and food and water intake were monitored. When the experiment was stopped, the mice were weighed and placed in their original cages.

### 2.12. In Vivo Body Composition Analysis

Time-Domain Nuclear Magnetic Resonance (TD-NMR) is a method based on the acquisition of radiofrequency signals generated by hydrogen spins from fluid and soft tissues, such as muscle and adipose tissue. The Minispec LF90 II was calibrated for mouse body composition measurements following ref. [32]. The measurement is non-invasive and does not require anesthesia or other preparation. Mice of 14 w were used for testing.

### 2.13. Statistical Analyses

All values are presented as the mean ± S.E.M. For statistical comparison of the experimental data, a robust nonparametric R ANOVA, fit linear mixed effect model, a two-way ANOVA, or a two-way RM ANOVA, was used with Sidak’s Post hoc test. A probability level of *p* < 0.05 was chosen as a threshold for statistical significance (*). A linear mixed model was applied with sex and genotype as main factors and body weight as a covariate.

## 3. Results

### 3.1. Generation of Gabra5^−/−^ Mouse Model

**Figure 1:**
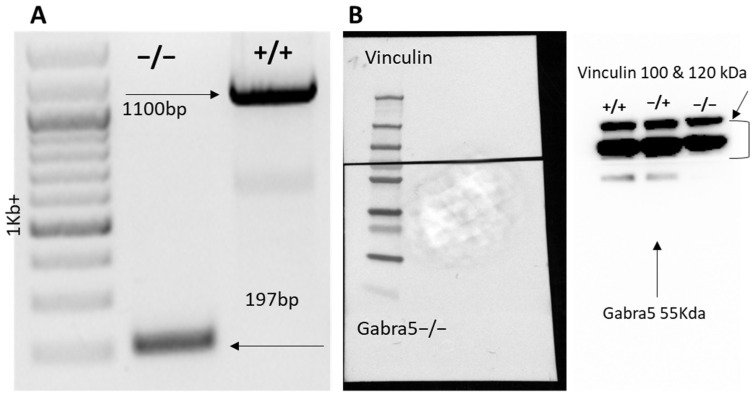
Model validation. (**A**) representative genotyping gel where the KO has a band of 197 bp and the WT 1100 bp. (**B**) western blot of WT, heterozygote and *Gabra5^−/−^* hippocampi. Colorimetric picture on the left and antibody bound protein exposed on the right. The α5 subunit protein was detected at 55 kDa in WTs and in the heterozygote, it was not observed in the homozygote. The loading control vinculin was detected at 100 and 120 kDa and seen in all samples.

The mouse line was generated through the CRISPR-based targeting of exon 3 deleting 903 nucleotides encompassing the crucial exon 3 of *Gabra5^−/−^* (Figure 1A). The elimination of the protein product was confirmed using a western blot of hippocampal lysates. The α5 protein was detected at its predicted size of 55 kDa in the WT control and in the heterozygote, and no detection was found in the homozygote hippocampus (Figure 1B). The loading control vinculin was used as it exceeds the α5 protein size with 50 kDa and is thus suitable for a full blot analysis as we would expect smaller truncated versions of the protein, if any expression was to be seen in mutants.

### 3.2. Gabra5^−/−^ Mice Exhibit Decreased Levels of Fecal Corticosterone Metabolite

**Figure 2:**
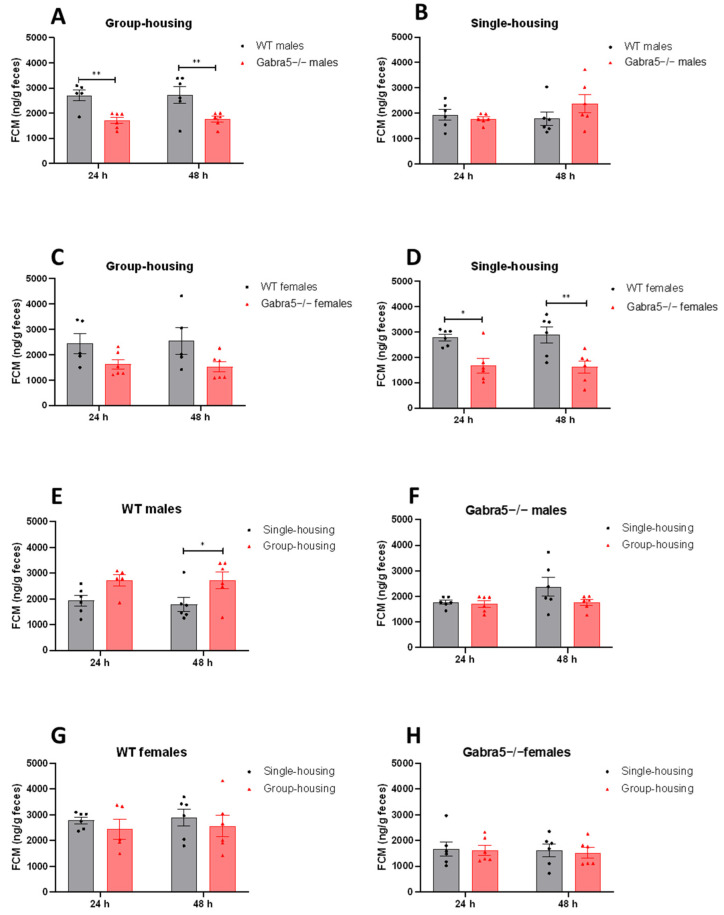
Fecal corticosterone metabolites concentration and effects of housing. (**A**–**D**) FCM concentrations depending on genotype within same housing-conditions. (**E**–**H**) Same FCM concentrations but depending on housing within sex and genotype. Two-way ANOVA with dependent measurements with Bonferroni’s post-hoc test, n = 6. All graphs were depicted with mean ± SEM. Significant effects of genotype or housing are indicated as * *p* < 0.05 and ** *p* < 0.01.

We were first interested in the stress levels of *Gabra5^−/−^* and control mice under regular home cage conditions, reflected by concentrations of fecal corticosterone metabolites (FCM). As an extension of this, we also compared FCM in mice that were either group- or single-housed over 24 and 48 h. Originally kept in groups of up to six individuals, the test animals were separated and kept in single-housed enclosures. The group-housed mice remained under these conditions as controls. We found that male *Gabra5^−/−^* mice had significantly lower FCM levels when group-housed collectively both times (main genotype effect *p* = 0.004; genotype/time interaction *p* > 0.05; Figure 2A; Supplementary Table S1). However, when single-housed *Gabra5^−/−^* males did not differ significantly from WT males (main genotype effect *p* = 0.49; genotype/time interaction *p* > 0.05; Figure 2B; Supplementary Table S1). In group-housed females, we could not detect significant differences in FCM between genotypes, but there was a trend towards lower FCM (main genotype effect *p* = 0.07; genotype/time interaction *p* > 0.05; Figure 2C; Supplementary Table S1). However, when the *Gabra5^−/−^* females were single-housed, FCM were significantly lower (main genotype effect *p* = 0.001; genotype/time interaction *p* > 0.05; Figure 2D; Supplementary Table S1).

Using the same data but looking at the effects of housing conditions on FCM levels within the same sex-genotype groups revealed that single-housing significantly decreased FCM concentrations in WT males, decreasing from a mean of 2715 to a mean of 1858 ng FCM per g feces (main housing effect *p* = 0.02; housing/time interaction *p* > 0.05: Figure 2E; Supplementary Table S1). In *Gabra5^−/−^* males and both WT and *Gabra5^−/−^* females, there were no statistically significant effects of housing on FCM concentrations (main genotype effects *p* = 0.18; 0.42; 0.81; Figure 2F–H; Supplementary Table S1). Time was not a significant factor for any of the sex-genotype combinations (main time effect *p* > 0.05; Figure 2).

The significant effect of housing detected in WT males’ corticosterone levels urged us to investigate whether the stress caused by group housing could be linked to aggression. Group housing could lead to more pronounced dominance behavior within groups of males, which could also affect testosterone levels in the blood. Therefore, we measured testosterone levels in single- and group-housed males 24 h and 48 h after separation, representing the state at once (24 h) and twice (48 h) handled and blood sampled. There was a significant increase in testosterone when handled twice for both genotypes in group-housed conditions (main time effect *p* = 0.009; time/genotype interaction *p* > 0.05; Supplementary Figure S1A). Time and handling did not affect the levels of testosterone in single-housed animals regardless of genotype (main time effect *p* = 0.58; Supplementary Figure S1B). Comparing the effect of housing within each genotype did not reveal significant changes with time; however, trends were observed for both WT and *Gabra5^−/−^* at 48 h (main housing effect *p* = 0.09; 0.07; Supplementary Figure S1C,D).

### 3.3. Open Field and Elevated Plus Maze Tests Do Not Suggest General Anxiety in Gabra5^−/−^ Mice

**Figure 3:**
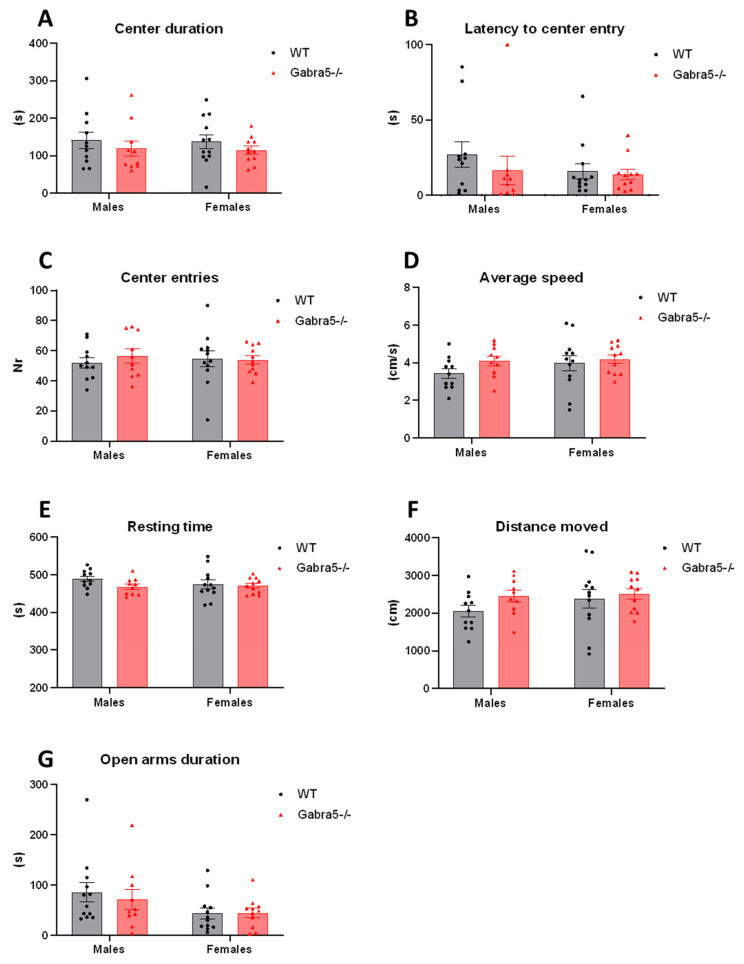
Open field and elevated plus maze tests. (**A**–**C**) OF anxiety-like test parameters; center duration, latency to enter center and number of center entries. (**D**–**F**) OF activity parameters; average speed, resting time and distance moved. (**G**) Elevated plus maze open arms duration. Two-way ANOVA main genotype effects all above *p* > 0.05. All figures are depicted with mean ± SEM, n = 12.

We performed more specialized behavioral testing similar to open field and elevated plus maze tests to investigate whether *Gabra5^−/−^* mice and WTs differ in general anxiety-like behavior. Based on the time spent in the aversive center, the latency to enter the center, the number of center entries, both supported and unsupported rears, we observed that *Gabra5^−/−^* mice do not exhibit anxiety-like tendencies (main genotype effect *p* = 0.23; *p* = 0.37; *p* = 0.65; *p* = 0.59; *p* = 0.39; genotype/sex interaction *p* > 0.05; Figure 3A–C, Supplementary Figure S2A,B) nor the average speed, total resting time, and total distance travelled differed (*p* = 0.16; 0.14; 0.16; genotype/sex interaction *p* > 0.05; Figure 3D–F). Time spent in the open arms in the elevated plus maze test did not significantly differ between the genotypes (*p* = 0.93; sex/genotype interaction *p* > 0.05; Figure 3G) nor did etiological parameters such as head dipping or rearing (main genotype effect *p* = 0.47; *p* = 0.95; genotype/sex interaction *p* > 0.05; Supplementary Figure S2C,D).

### 3.4. Gabra5^−/−^ Animals Do Not Differ in Contextual and Delayed Cued Fear Conditioning

We tested the mice in delayed cued fear conditioning and contextual fear conditioning to evaluate possible phenotypes regarding learning and memory.

**Figure 4:**
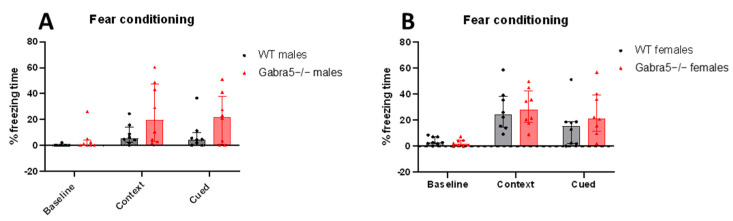
Contextual and delayed cued fear conditioning. (**A**,**B**) Baseline, contextual and cued- freezing time is depicted in percentage. No significant differences in the contextual and cued parameters between WT and *Gabra5^−/−^* animals for both sexes. Fit linear Mixed Effect Model main genotype effect *p* > 0.05. All figures are depicted with median with interquartile range, n = 8.

Based on our observations, we report no significant difference in freezing time between WT mice and *Gabra5^−/−^* in the contextual and delayed cued probe trial (main genotype effect *p* = 0.06; genotype/sex interaction *p* > 0.05; genotype/trial interaction *p* > 0.05; Figure 4A,B).

### 3.5. Gabra5^−/−^ Animals Exhibit Decreased Rearing Behavior

As an approach to monitoring specific behavioral domains, such as drinking and feeding behavior, locomotor activity, and metabolic rate, animals were placed into calorimetric cages. These parameters cover behavioral and metabolic functions in rodents that are affected by corticosterone [33,34,35]. While rearing has been a measure of activity, it has also been proven to be valuable in terms of evaluating emotionality, such as anxiety and fear, thus making it an interesting behavior for us to evaluate [34].

**Figure 5:**
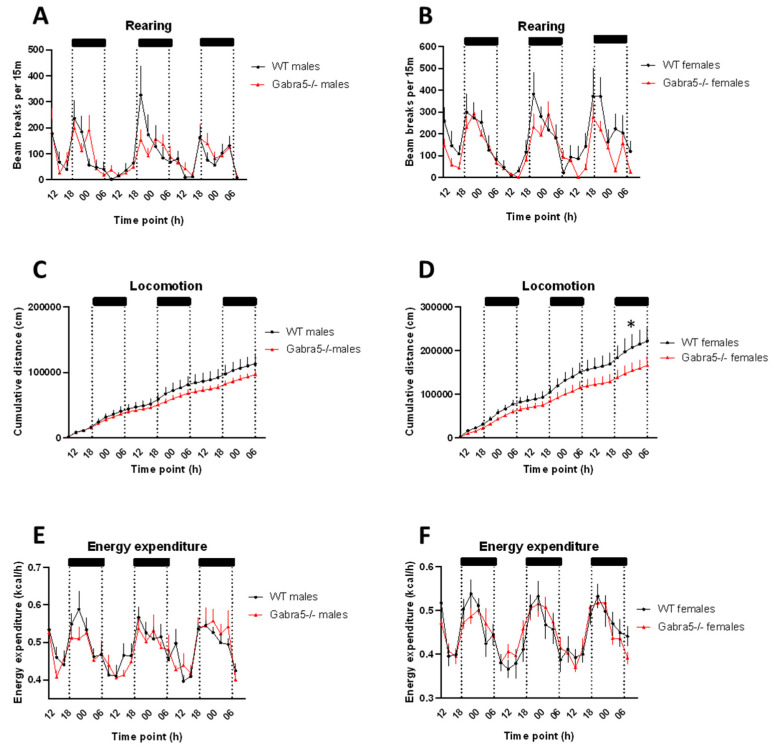
Indirect calorimetry- Rearing, locomotion, and energy expenditure. (**A**,**B**) rearing was decreased during the dark phases (shaded) in KO animals. (**C**,**D**) a tendency of decreased locomotion was observed in *Gabra5^−/−^* animals of both sexes however significant for females. (**E**,**F**) Energy expenditure was not significantly altered for either sex. Regression analysis, n = 8. All figures depict mean ± SEM. Significant effects of genotype is indicated as * *p* < 0.05.

*Gabra5^−/−^* mice exhibited significantly fewer rearings per time interval during the three combined dark phases (main genotype effect *p* = 0.04; Figure 5A,B). Total locomotion for both sexes was not significantly altered but trended to be decreased over the cumulative period (main genotype effect *p* = 0.07; Figure 5C,D). Additional analysis showed that *Gabra5^−/−^* females moved significantly less than control animals (female genotype effect *p* = 0.04; Figure 5C,D) which was not detected in males (main genotype effect *p* = 0.82; Figure 5C,D). Energy expenditure was not significantly different by gender (main genotype effect *p* = 0.96; Figure 5E,F). Food and water intake were also monitored. The animals did not differ in food intake (*p* = 0.56; Supplementary Figure S2A,B), but *Gabra5^−/−^* males drank significantly less than controls (*p* = 0.0003; Supplementary Figure S2C,D).

Body composition was measured to investigate if small differences in energy fluxes affect endogenously stored energy in white adipose tissue or result in a lean phenotype. However, there were no significant differences in the relationship between fat and lean mass when regressed versus bodyweight (genotype effect on slope and intercept *p* > 0.05; Supplementary Figure S3A–D).

### 3.6. Hippocampal CA1 Pyramidal Neurons Exhibit Reduced Excitability in Gabra5^−/−^ Mice

**Figure 6:**
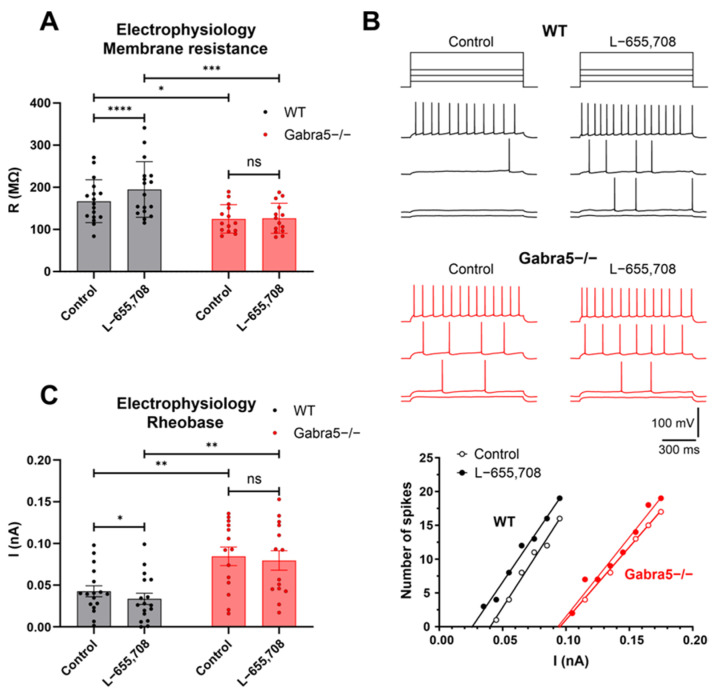
Deletion of *Gabra5* reduces neuronal excitability in CA1 hippocampal slices. (**A**) Comparison of membrane resistance (R) of CA1 pyramidal neurons from WT and *Gabra5^−/−^* mice in the absence or presence of L-655,708. Lower R values indicate reduced neuronal excitability in *Gabra5^−/−^* mice. L-655,708, an inverse agonist of GABA(A) receptors containing the α5 subunit, significantly reduced membrane resistance of neurons in WT but not in *Gabra5^−/−^* mice. Values are mean ± SEM of 17 (WT) and 14 (*Gabra5^−/−^*) experiments. **** *p* < 0.0001; two-way RM ANOVA followed by Sidak’s multiple comparisons test was used to compare R values for the same neurons in the absence (Control) and presence of L-655,708. *** *p* < 0.05, * *p* < 0.05; Two-way ANOVA followed by Sidak’s multiple comparisons test was used to compare R values in WT and *Gabra5^−/−^* neurons. (**B**) Example traces showing voltage responses of WT (black) and *Gabra5^−/−^* (red) pyramidal neurons evoked by current step stimulations (shown above the traces) of amplitude 25, 35, 45 and 85 pA (WT) or 95, 105, 115 and 155 pA (*Gabra5^−/−^*) in the absence (Control) or presence of L-655,708. The plots below show spike frequency as a function of injected current for the same neurons as above. The minimum current required to elicit an action potential (rheobase) was determined by fitting the dependence to a linear function. Note that the *Gabra5^−/−^* neuron requires stronger current stimulation to elicit somatic action potentials and that L-655,708 reduced the threshold stimulus amplitude in the WT but not in the *Gabra5^−/−^* neuron. (**C**) Mean rheobase values for WT and *Gabra5^−/−^* neurons in the absence (Control) or presence of L-655,708. In contrast to WT neurons, *Gabra5^−/−^* neurons show significantly increased rheobase independent of the presence of L-655,708. Values are mean ± SEM of 17 (WT) and 14 (*Gabra5^−/−^*) experiments. * *p* < 0.05; two-way RM ANOVA followed by Sidak’s multiple comparisons test was used to compare rheobase for the same neurons in the absence (Control) and presence of L-655,708. ** *p* < 0.01; Two-way ANOVA followed by Sidak’s multiple comparisons test was used to compare rheobase in WT and *Gabra5^−/−^* neurons.

To test whether genetic ablation of *Gabra5* leads to functional changes in central neurons, we examined the electrical properties of pyramidal cells in acutely isolated live CA1 hippocampal slices using the patch-clamp technique. These cells typically express α5 receptors at both synaptic and extrasynaptic sites, where they mediate tonic GABAergic inhibition [26,31,36]. Compared with WT neurons, *Gabra5^−/−^* neurons exhibited a more negative resting membrane potential (−76.8 ± 1.0 mV, n = 17 vs. −81.3 ± 1.3 mV, n = 14; *p* = 0.0193, two-way ANOVA followed by Sidak’s multiple comparison test) and significantly lower membrane resistance values (Figure 6A). These rather unexpected observations indicated reduced excitability of hippocampal neurons lacking the GABA(A) receptor α5 subunit and suggested the presence of tonic hyperpolarizing conductance. Membrane potential and resistance of WT neurons were significantly increased in the presence of L-655,708, a specific inverse agonist of GABA(A)α5-containing receptors [12], by 1.0 ± 0.3 mV and 27.8 ± 6.1 MΩ, respectively (*p* = 0.0015 and *p* ˂ 0.0001, respectively; two-way RM ANOVA with Sidak’s multiple comparison test), whereas these parameters were insensitive to the drug in *Gabra5^−/−^* neurons (Figure 6A; *p* = 0.7668 and 0.9383, respectively). Thus, the tonic hyperpolarizing conductance in *Gabra5^−/−^* neurons did not appear to be affected by L-655,708, suggesting it was not mediated by α5 subunit-containing GABA(A) receptors. Consistent with the presence of increased hyperpolarizing and shunting inhibition in *Gabra5^−/−^* neurons, we observed a significant increase in their rheobase values for action potentials evoked by long depolarizing stimuli (from 0.043 ± 0.007 nA to 0.084 ± 0.011 nA, *p* = 0.003, two-way ANOVA followed by Sidak’s multiple comparison test, n = 17 WT and 14 *Gabra5^−/−^* neurons) (Figure 6B,C). L-655,708 significantly reduced rheobase values in WT neurons (to 0.033 ± 0.007 nA, *p* = 0.049, two-way RM ANOVA followed by Sidak’s multiple comparison test, n = 17) but not in *Gabra5^−/−^* neurons (to 0.079 ± 0.012 nA, *p* = 0.435, two-way RM ANOVA followed by Sidak’s multiple comparison test, n = 14) (Figure 6B,C). Taken together, the effects of L-655,708 on the electrical properties of WT neurons are consistent with the presence of α5 subunit-containing GABA(A) receptors mediating tonic GABAergic inhibition in CA1 pyramidal neurons [26,31,37]. In contrast, the absence of L-655,708 effects in *Gabra5^−/−^* neurons demonstrates the absence of the receptors in these neurons, leading to a significant change in their functional properties, presumably through homeostatic upregulation of distinct ion channels.

## 4. Discussion

*GABRA5* is a gene frequently missing from the maternal chromosome in Angelman syndrome patients that harbor a large deletion ranging from 4–6 Mb [38]. We generated a new mouse model deficient for *Gabra5* by targeting the critical exon 3 of the *Gabra5* gene with the CRISPR/Cas9 technique.

The model was used to evaluate how GABA(A)R influences behavioral anxiety-like behavior in correlation to corticosterone levels. Our study revealed significantly lower fecal corticosterone metabolites in the *Gabra5^−/−^* mice and decreased anxiety-like behavior exhibited by decreased rearing behavior.

Regarding the effect of functional *Gabra5* ablation and its neurobehavioral effects, we assayed corticosterone in feces (in single- and group-housed conditions twice during a period of 24 h). Measuring FCM and not plasma corticosterone offers an advantage as it is less invasive to the animal and the confounding effect that stress of blood sampling may have on corticosterone is eliminated using this methodology. Furthermore, it offers an advantage in evaluating corticosterone when handling larger groups of animals as it is not subjected to the rapid increase in corticosterone levels due to the response of a stressor, which can be increased as fast as within 2–5 min [39]. The *Gabra5^−/−^* males exhibited lower FCM levels in group-housed conditions, but when single-housed, the significant difference disappeared. *Gabra5^−/−^* females had significantly less secreted FCM when single-housed but no significance appeared in FCM levels in group-housed conditions although a tendency could be observed, suggesting a lower baseline of FCM in *Gabra5^−/−^* females. This could point towards a decrease in experienced anxiety. Interestingly, we found that the housing-conditions only had a significant effect on FCM levels in WT males but not in any of the other sex-genotype groups.

Male mice can exhibit aggression towards one another when group-housed, which would lead to higher FCM levels under such group-housed conditions [40]. As testosterone is linked to aggressive behavior [41] we found that testosterone concentration in males in the same setup as the FCM test is increased at the second time of handling in group-housed males irrespective of the genotype. As there were no significant differences between *Gabra5^−/−^* and WT males, differences in aggression is likely not the cause of lower FCM levels in *Gabra5^−/−^* males.

Based on reports from patients with impairments in the *GABRA5* gene and animal studies with allosteric modulators to the α5 subunit, we were expecting to see an increase in anxiety-like behavior in our *Gabra5^−/−^* model [15,42].

However, the open field and elevated plus maze tests could not detect an anxiety phenotype in the *Gabra5^−/−^* animals.

Another aspect we followed is rearing behavior that is largely hippocampal-dependent. as its formation is a pivotal component of the neural structure responsible for anxiety [43]. Rearing is a phenomenon when four-legged animals stand on their hind legs and explore their environment, which is a common response to novelty in rodents [43]. It has been suggested that the benefits of rearing in terms of information gathering are traded off by the risks of exploration. In a study by Blanchard and Blanchard [44] it was found that anxiolytics decreased rearing in situations interpretable as eliciting anxiety in the subjects [44]. In the Starcase test, the number of rearing behaviors is observed, and an increase is interpreted as anxiety-like behavior [45]. However, Sturman with colleagues demonstrated rears attenuation in open field as a result of acute restrain or swim stress but also handling and less angiogenic setup context [34]. These results suggest a U-relationship between rearing and stress level. Based on this, one would argue that rearing occurs the least during low or extreme levels of anxiety. Additionally, studies have shown that rearing, albeit initially considered a parameter for assessing locomotion or activity, is valuable for interpreting the emotionality of the animal, such as anxiety and fear [34]. Rearing was highly influenced by external stress-evoking factors such as noise and light exposure, clearly showing the suitability of employing rearing behavior as a measurement of experienced anxiety [34].

We identified decreased rearing in our *Gabra5^−/−^* animals during the dark phase of the day, the period when the animals are active, suggesting lower levels of anxiety in a new environment. However, rearing in the stressful EPM and OF tests was not significantly different. One needs to keep in mind that the OF and EPM have a much shorter duration of only up to 10 min in a stress-evoking environment, whereas the calorimetric setup runs over 72 h in a less stressful home cage environment.

General locomotion was only reduced in *Gabra5^−/−^* females, however, not in males, and energy expenditure and feeding did not differ in any sex, thus the reduced rearing is not a result of other possible confounding factors.

That the activation of GABA receptors has an anxiolytic effect is well known, and agonists such as benzodiazepines have been prescribed for anxiety-disorders for decades [46]. However, the contribution of individual subunits to the anxiolytic effect of the receptor is still being deduced by pharmacological inhibition and knock-out mouse studies. Based on increasing evidence of the anxiolytic effect of the α5 subunit, such as activation of it leading to increased time spent in the anxiogenic center in EPM [16,18], we were expecting to see increased anxiety-like behavior in the EPM and OF tests. The fact that we did not observe significant differences between the genotypes in those tests, but moreover, the *Gabra5^−/−^* exhibited significantly less rearing behavior in other experimental setups, suggests that there might be a developmental adaptation (functional compensation), found occasionally in constitutive KO mice [47].

A gold standard test to evaluate learning and memory, both hippocampal- dependent and -independent, are the contextual and delayed cued fear conditioning [29]. As the α5 protein is highly expressed in the hippocampus, one may expect to see effects in the freezing period following the context or cue for the aversive stimulus. We did not observe an effect of the α5 ablation in these two parameters. This is in line with previous studied models based on *Gabra5* manipulated animals [48,49]. However, the trace fear conditioning was shown to involve the hippocampal α5 subunit [48], which would have been a valuable addition to the methodology along with contextual fear conditioning to test hippocampal-dependent memory. Worth noting is that the fear conditioning tests are also largely dependent on the amygdala, which makes the test less straightforward to interpret.

Finally, genetic ablation of *Gabra5* showed to have a significant impact on the excitability of CA1 pyramidal neurons. These cells in *Gabra5^−/−^* mice exhibited shifted resting membrane potentials toward more hyperpolarized values, lower input resistance, and increased action potential rheobase. Our findings thus suggest that, in this model, the loss of tonic inhibition mediated by α5-containing extrasynaptic GABA(A) receptors [26,31,36] led to the generation of a different compensatory tonic inhibitory conductance by CA1 pyramidal neurons. Previous work has revealed homeostatic relationships between the expression of *Gabra5* and other GABA(A) receptor subunits or the hyperpolarization-activated cyclic nucleotide-gated (HCN) channels [50,51]. Given the hyperpolarizing effect of the shunt conductance and the value of the chloride equilibrium potential under our experimental conditions (~38 mV), we hypothesize that *Gabra5^−/−^* neurons could also homeostatically increase the expression of some tonically active K+ channels, such as the two-pore domain K+ channel TASK-1 [52]. Further experiments will be required to determine the mechanisms underlying tonic inhibitory conductance in CA1 pyramidal neurons of our *Gabra5^−/−^* mice.

Our findings differ from previous experiments performed on a very similar model also encompassing a deletion of exon 3, where the authors reported a reduced tonic inhibitory conductance and increased excitability of principal neurons in the hippocampus [27,37]. In a later study using the same mouse model on a C57BL/6J and Sv129Ev background, they described that the activity of α5 containing receptors increases the threshold for the induction of long-term potentiation at stimulation frequencies of 10–20 Hz [30]. This was independent from synaptic transmission, indeed in line with the mainly extrasynaptic localization of the α5 subunit. However, the deletion model or pharmacological inhibition of α5GABAARs caused no change in baseline membrane potential or input resistance [30]. Based on the similarity of the models and the mouse backgrounds used, there is no apparent reason for the differences observed in the electrophysiological recordings. However, the models were generated by different means of transgenesis. The model by Collinson et al. (2002) [27] was produced via a neocassette, substituting exon 3, whereas our model was CRISPR-mediated, producing a genetically “cleaner” model. Moreover, the insertion of neocassettes has been shown to influence metabolism and genes in the vicinity of the insertion [53].

We believe that the reduced excitability in our recordings might be a consequence of functional compensation, in line with the discussion regarding anxiety-like behavior in our *Gabra5^−/−^* model. An adaptation that perhaps was not developed in the previous mouse model [27]

In this work we studied effects of the α5 subunit ablation on behavior connected to anxiety and stress, linked to corticosterone concentration. Based on the electrophysiological recordings and lack of phenotype in hippocampal-dependent learning and memory, we assume that the functional ablation of the *Gabra5* gene leads to functional compensation with other channels or GABA receptor subunits in this model.

## Data Availability

The datasets used and/or analyzed during the current study are available from the corresponding author on reasonable request.

## Supplementary Materials

The following supporting information can be downloaded at: https://www.mdpi.com/article/10.3390/genes14020285/s1, Figure S1. Testosterone levels in single- and group-housed males, Figure S2. Rearing and head dipping. Figure S3. Indirect calorimetry food and water intake, Figure S4. Body composition analyses, Supplementary Table S1. Concentration of fecal corticosterone metabolites.

## References

[1] Kagias K., Nehammer C., Pocock R. (2012). Neuronal Responses to Physiological Stress. Front. Genet..

[2] Goodnite P.M. (2014). Stress: A concept analysis. Nurs. Forum.

[3] Hill J.W. (2012). PVN pathways controlling energy homeostasis. Indian J. Endocrinol. Metab..

[4] Smith S.M., Vale W.W. (2006). The role of the hypothalamic-pituitary-adrenal axis in neuroendocrine responses to stress. Dialogues Clin. Neurosci..

[5] Jubb A.W., Boyle S., Hume D.A., Bickmore W.A. (2017). Glucocorticoid Receptor Binding Induces Rapid and Prolonged Large-Scale Chromatin Decompaction at Multiple Target Loci. Cell Rep..

[6] Raglan G.B., Schmidt L.A., Schulkin J. (2017). The role of glucocorticoids and corticotropin-releasing hormone regulation on anxiety symptoms and response to treatment. Endocr. Connect..

[7] Sapolsky R.M. (2004). Why Zebras Don’t Get Ulcers: The Acclaimed Guide to Stress, Stress-Related Diseases, and Coping.

[8] Cullinan W.E., Ziegler D.R., Herman J.P. (2008). Functional role of local GABAergic influences on the HPA axis. Brain Struct. Funct..

[9] Gunn B., Brown A.R., Lambert J.J., Belelli D. (2011). Neurosteroids and GABAA Receptor Interactions: A Focus on Stress. Front. Neurosci..

[10] Cullinan W.E., Helmreich D.L., Watson S.J. (1996). Fos expression in forebrain afferents to the hypothalamic paraventricular nucleus following swim stress. J. Comp. Neurol..

[11] Everington E.A., Gibbard A.G., Swinny J.D., Seifi M. (2018). Molecular Characterization of GABA-A Receptor Subunit Diversity within Major Peripheral Organs and Their Plasticity in Response to Early Life Psychosocial Stress. Front. Mol. Neurosci..

[12] Jacob T.C. (2019). Neurobiology and Therapeutic Potential of α5-GABA Type A Receptors. Front. Mol. Neurosci..

[13] Sarkar J., Wakefield S., MacKenzie G., Moss S.J., Maguire J. (2011). Neurosteroidogenesis is required for the physiological response to stress: Role of neurosteroid-sensitive GABAA receptors. J. Neurosci..

[14] Bird L.M. (2014). Angelman syndrome: Review of clinical and molecular aspects. Appl. Clin. Genet..

[15] Hodges L.M., Fyer A.J., Weissman M.M., Logue M.W., Haghighi F., Evgrafov O., Rotondo A., Knowles J.A., Hamilton S.P. (2014). Evidence for linkage and association of GABRB3 and GABRA5 to panic disorder. Neuropsychopharmacology.

[16] Magnin E., Francavilla R., Amalyan S., Gervais E., David L.S., Luo X., Topolnik L. (2019). Input-Specific Synaptic Location and Function of the α5 GABAA Receptor Subunit in the Mouse CA1 Hippocampal Neurons. J. Neurosci..

[17] Navarro J.F., Burón E., Martın-López M. (2002). Anxiogenic-like activity of L-655,708, a selective ligand for the benzodiazepine site of GABA(A) receptors which contain the α-5 subunit, in the elevated plus-maze test. Prog. Neuro Psychopharmacol. Biol. Psychiatry.

[18] Behlke L.M., Foster R.A., Liu J., Benke D., Benham R.S., Nathanson A.J., Yee B.K., Zeilhofer H.U., Engin E., Rudolph U. (2016). A Pharmacogenetic ‘Restriction-of-Function’ Approach Reveals Evidence for Anxiolytic-Like Actions Mediated by α5-Containing GABAA Receptors in Mice. Neuropsychopharmacology.

[19] Olsen R.W., Sieghart W. (2009). GABA A receptors: Subtypes provide diversity of function and pharmacology. Neuropharmacology.

[20] Myers B., McKlveen J.M., Herman J.P. (2014). Glucocorticoid actions on synapses, circuits, and behavior: Implications for the energetics of stress. Front. Neuroendocrinol..

[21] Martin L.J., Bonin R.P., Orser B.A. (2009). The physiological properties and therapeutic potential of alpha5-GABAA receptors. Biochem. Soc. Trans..

[22] Shin L.M., Liberzon I. (2010). The neurocircuitry of fear, stress, and anxiety disorders. Neuropsychopharmacol. Off. Publ. Am. Coll. Neuropsychopharmacol..

[23] Herman J.P., McKlveen J.M., Ghosal S., Kopp B., Wulsin A., Makinson R., Scheimann J., Myers B. (2016). Regulation of the Hypothalamic-Pituitary-Adrenocortical Stress Response. Compr. Physiol..

[24] Cominski T.P., Jiao X., Catuzzi J.E., Stewart A.L., Pang K.C.H. (2014). The role of the hippocampus in avoidance learning and anxiety vulnerability. Front. Behav. Neurosci..

[25] Jimenez J.C., Su K., Goldberg A.R., Luna V.M., Biane J.S., Ordek G., Zhou P., Ong S.K., Wright M.A., Zweifel L. (2018). Anxiety Cells in a Hippocampal-Hypothalamic Circuit. Neuron.

[26] Caraiscos V.B., Elliott E.M., You-Ten K.E., Cheng V.Y., Belelli D., Newell J.G., Jackson M.F., Lambert J.J., Rosahl T.W., Wafford K.A. (2004). Tonic inhibition in mouse hippocampal CA1 pyramidal neurons is mediated by alpha5 subunit-containing γ-aminobutyric acid type A receptors. Proc. Natl. Acad. Sci. USA.

[27] Collinson N., Kuenzi F.M., Jarolimek W., Maubach K.A., Cothliff R., Sur C., Smith A., Otu F.M., Howell O., Atack J.R. (2002). Enhanced learning and memory and altered GABAergic synaptic transmission in mice lacking the α 5 subunit of the GABAA receptor. J. Neurosci..

[28] Kulesskaya N., Voikar V. (2014). Assessment of mouse anxiety-like behavior in the light-dark box and open-field arena: Role of equipment and procedure. Physiol. Behav..

[29] Shoji H., Takao K., Hattori S., Miyakawa T. (2014). Contextual and cued fear conditioning test using a video analyzing system in mice. J. Vis. Exp..

[30] Martin L.J., Zurek A.A., MacDonald J.F., Roder J.C., Jackson M.F., Orser B.A. (2010). α5GABAAReceptor Activity Sets the Threshold for Long-Term Potentiation and Constrains Hippocampus-Dependent Memory. J. Neurosci..

[31] Glykys J., Mody I. (2006). Hippocampal network hyperactivity after selective reduction of tonic inhibition in GABA A receptor alpha5 subunit-deficient mice. J. Neurophysiol..

[32] Morla L., Shore O., Lynch I.J., Merritt M.E., Wingo C.S. (2020). A noninvasive method to study the evolution of extracellular fluid volume in mice using time-domain nuclear magnetic resonance. Am. J. Physiol. Ren. Physiol..

[33] Mitra R., Sapolsky R.M. (2008). Acute corticosterone treatment is sufficient to induce anxiety and amygdaloid dendritic hypertrophy. Proc. Natl. Acad. Sci. USA.

[34] Sturman O., Germain P.-L., Bohacek J. (2018). Exploratory rearing: A context- and stress-sensitive behavior recorded in the open-field test. Stress.

[35] Izzi-Engbeaya C., Ma Y., Buckley N.W., Ratnasabapathy R., Richardson E., Counsell J.R., Fernandes-Freitas I., Norton M., Farooq G., Mirza Z. (2020). Effects of corticosterone within the hypothalamic arcuate nucleus on food intake and body weight in male rats. Mol. Metab..

[36] Serwanski D.R., Miralles C.P., Christie S.B., Mehta A.K., Li X., De Blas A.L. (2006). Synaptic and nonsynaptic localization of GABAA receptors containing the alpha5 subunit in the rat brain. J. Comp. Neurol..

[37] Bonin R.P., Martin L.J., MacDonald J.F., Orser B.A. (2007). Alpha5GABAA receptors regulate the intrinsic excitability of mouse hippocampal pyramidal neurons. J. Neurophysiol..

[38] Syding L.A., Nickl P., Kasparek P., Sedlacek R. (2020). CRISPR/Cas9 Epigenome Editing Potential for Rare Imprinting Diseases: A Review. Cells.

[39] Gasser P.J., Lowry C.A., Orchinik M., Pfaff D.W., Joels M. (2009). 41—Rapid corticosteroid actions on behavior: Mechanisms and implications. Hormones, Brain and Behavior.

[40] Lidster K., Owen K., Browne W.J., Prescott M.J. (2019). Cage aggression in group-housed laboratory male mice: An international data crowdsourcing project. Sci. Rep..

[41] Giammanco M., Tabacchi G., Giammanco S., Di Majo D., La Guardia M. (2005). Testosterone and aggressiveness. Med. Sci. Monit. Int. Med. J. Exp. Clin. Res..

[42] Piantadosi S.C., French B.J., Poe M.M., Timić T., Marković B.D., Pabba M., Seney M.L., Oh H., Orser B.A., Savić M.M. (2016). Sex-Dependent Anti-Stress Effect of an α5 Subunit Containing GABAA Receptor Positive Allosteric Modulator. Front. Pharmacol..

[43] Lever C., Burton S., O’Keefe J. (2006). Rearing on hind legs, environmental novelty, and the hippocampal formation. Rev. Neurosci..

[44] Blanchard D.C., Blanchard R.J., Rodgers R.J., Olivier B., Mos J., Slangen J.L. (1991). Risk Assessment and animal models of anxiety. Animal Models in Psychopharmacology.

[45] Cryan J.F., Sweeney F.F. (2011). The age of anxiety: Role of animal models of anxiolytic action in drug discovery. Br. J. Pharmacol..

[46] Balon R., Starcevic V. (2020). Role of Benzodiazepines in Anxiety Disorders. Anxiety Disord..

[47] El-Brolosy M.A., Stainier D.Y.R. (2017). Genetic compensation: A phenomenon in search of mechanisms. PLoS Genet..

[48] Crestani F., Keist R., Fritschy J.-M., Benke D., Vogt K., Prut L., Bluthmann H., Mohler H., Rudolph H. (2002). Trace fear conditioning involves hippocampal alpha5 GABA(A) receptors. Proc. Natl. Acad. Sci. USA.

[49] Martin L.J., Oh G.H.T., Orser B.A. (2009). Etomidate targets alpha5 γ-aminobutyric acid subtype A receptors to regulate synaptic plasticity and memory blockade. Anesthesiology.

[50] Bonin R.P., Zurek A.A., Yu J., Bayliss D.A., Orser B.A. (2013). Hyperpolarization-Activated Current (Ih) Is Reduced in Hippocampal Neurons from Gabra5^−/−^ Mice. PLoS ONE.

[51] Glykys J., Mann E.O., Mody I. (2008). Which GABA(A) receptor subunits are necessary for tonic inhibition in the hippocampus?. J. Neurosci..

[52] Brickley S.G., Revilla V., Cull-Candy S.G., Wisden W., Farrant M. (2001). Adaptive regulation of neuronal excitability by a voltage-independent potassium conductance. Nature.

[53] Jin C., Kang H., Yoo T., Ryu J.R., Yoo Y.-E., Ma R., Zhang Y., Kang H.R., Kim Y., Seong H. (2021). The Neomycin Resistance Cassette in the Targeted Allele of Shank3B Knock-Out Mice Has Potential Off-Target Effects to Produce an Unusual Shank3 Isoform. Front. Mol. Neurosci..

