# Supplementary material for: Ablation of Gabra5 Influences Corticosterone Levels and Anxiety-like Behavior in Mice
Source: Genes (Basel). 2023 Jan 21;14(2):285. doi: 10.3390/genes14020285 (PMC9956889; doi:10.3390/genes14020285)
Supplement: Supplementary file 1 [file genes-14-00285-s001.zip › Figure S1. Testosterone_housing.pdf]

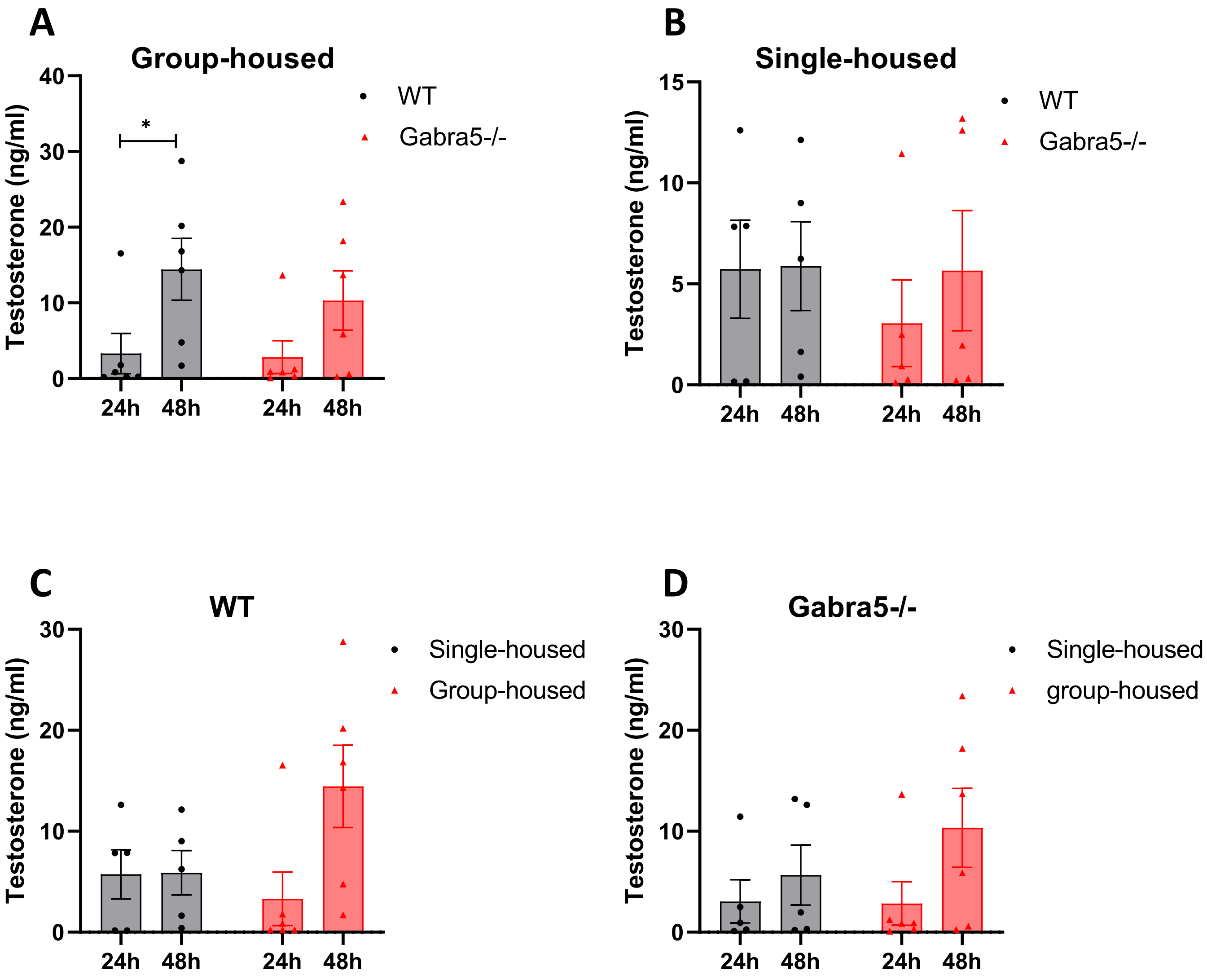

Figure S1. Testosterone levels in single- and group-housed males. A-B testosterone levels comparison between genotypes in group- and single-housing. C-D Same testosterone values but depending on housing within genotype. Two-way ANOVA with dependent measurements with Bonferroni's post-hoc test, n=6. Graphs show mean ± SEM. Significant effects of genotype or housing are indicated as *$p < 0.05$.
