# Supplementary material for: Ablation of Gabra5 Influences Corticosterone Levels and Anxiety-like Behavior in Mice
Source: Genes (Basel). 2023 Jan 21;14(2):285. doi: 10.3390/genes14020285 (PMC9956889; doi:10.3390/genes14020285)
Supplement: Supplementary file 1 [file genes-14-00285-s001.zip › Figure S3. Food_water_intake.pdf]

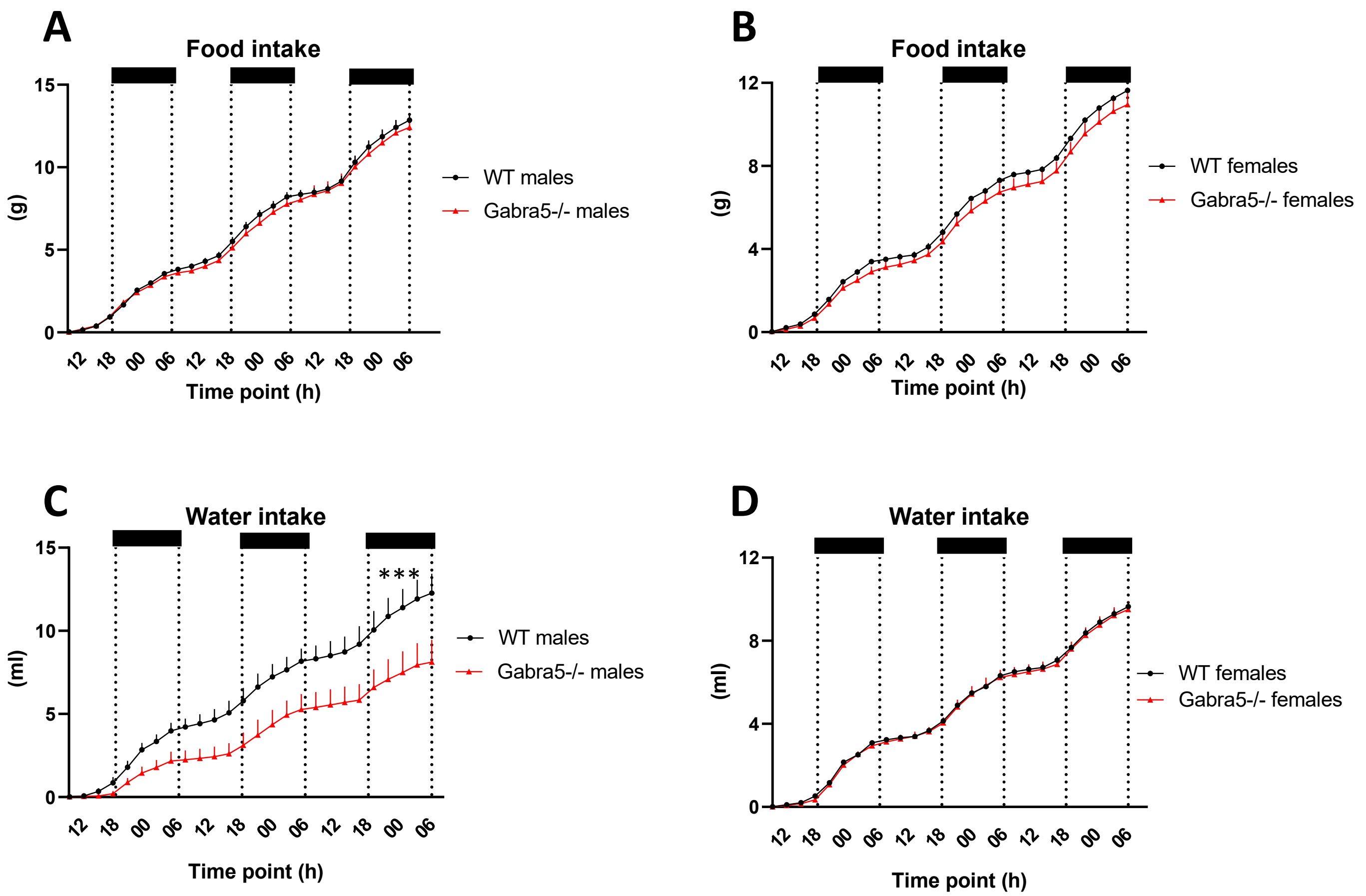

Figure S3. Indirect calorimetry food and water intake. AB Food intake monitored over three consecutive days where shaded areas represent dark periods. CD Water intake monitored over three consecutive days where shaded areas represent dark periods
