# Supplementary material for: Ablation of Gabra5 Influences Corticosterone Levels and Anxiety-like Behavior in Mice
Source: Genes (Basel). 2023 Jan 21;14(2):285. doi: 10.3390/genes14020285 (PMC9956889; doi:10.3390/genes14020285)
Supplement: Supplementary file 1 [file genes-14-00285-s001.zip › Figure S4. Body composition.pdf]

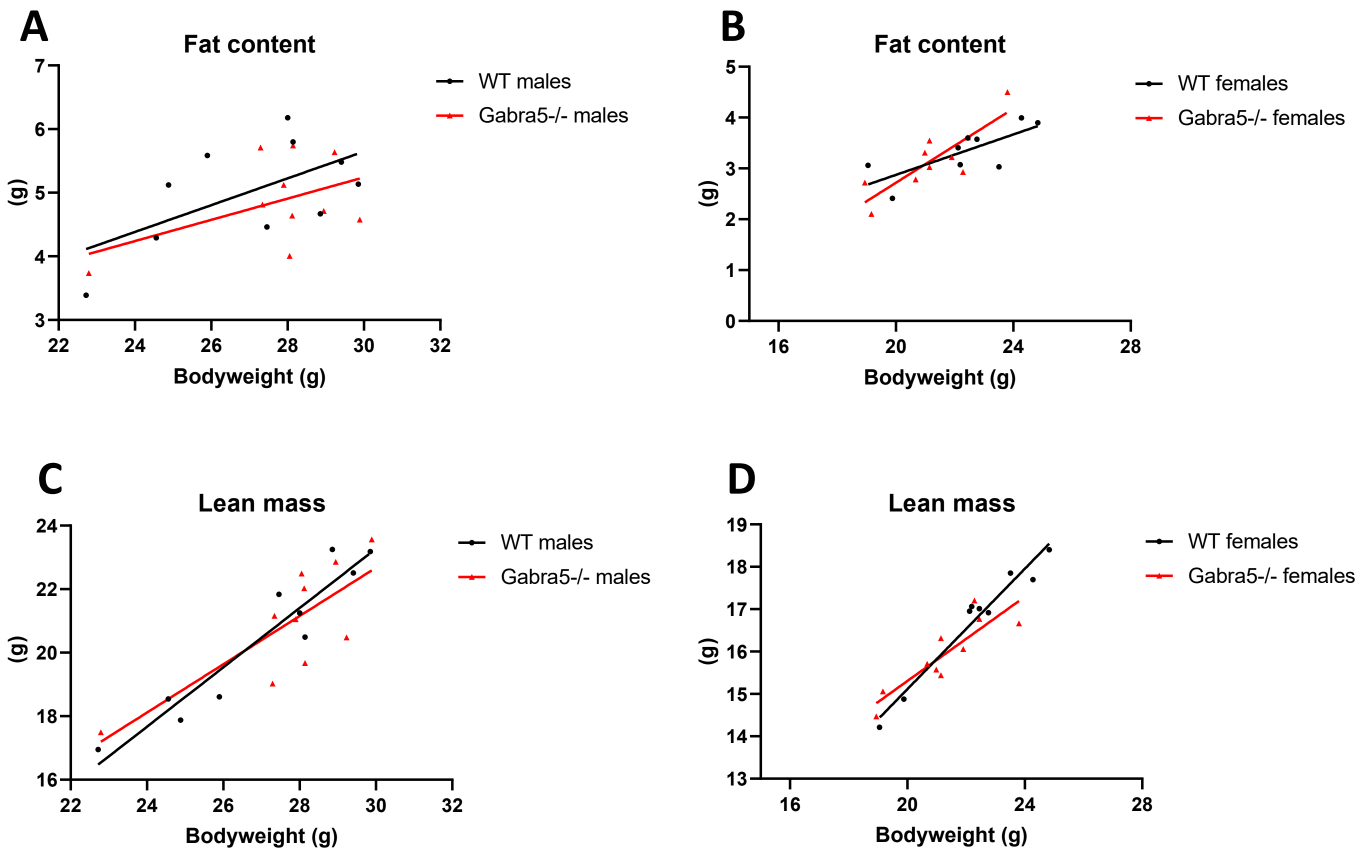

Figure S4. Body composition analyses. AD Fat and lean body mass plotted over bodyweight in WT and Gabra5 -/- animals. The body composition was measure with Time domain – Nuclear Magnetic resonance (TD-NMR), a method based on the acquisition of radiofrequency signals generated by hydrogen spins from fluid and soft tissues, such as muscle and adipose tissue. The Minispec LF90 II was calibrated for mice body composition measurements. The measurement is non-invasive and does not require anesthesia or other preparation.
