# Supplementary material for: Ablation of Gabra5 Influences Corticosterone Levels and Anxiety-like Behavior in Mice
Source: Genes (Basel). 2023 Jan 21;14(2):285. doi: 10.3390/genes14020285 (PMC9956889; doi:10.3390/genes14020285)
Supplement: Supplementary file 1 [file genes-14-00285-s001.zip › Table S1. Concentration of fecal corticosterone metabolites.pdf]

	Males		Females	
**Housing-condition**	**WT**	**Gabra5$^{-/-}$**	**WT**	**Gabra5$^{-/-}$**
Group	2715 ± 196	1735 ± 81	2505 ± 276	1572 ± 134
Single	1858 ± 162	2076 ± 197	2836 ± 165	1644 ± 178

Table S1. Concentration of fecal corticosterone metabolites. The values are presented as ng/ml as mean ± SEM in group- and single-housed conditions. All days pooled.
