# Supplementary material for: Ablation of Gabra5 Influences Corticosterone Levels and Anxiety-like Behavior in Mice
Source: Genes (Basel). 2023 Jan 21;14(2):285. doi: 10.3390/genes14020285 (PMC9956889; doi:10.3390/genes14020285)
Supplement: Supplementary file 1 [file genes-14-00285-s001.zip › Figure S2. Rearing and head dipping.pdf]

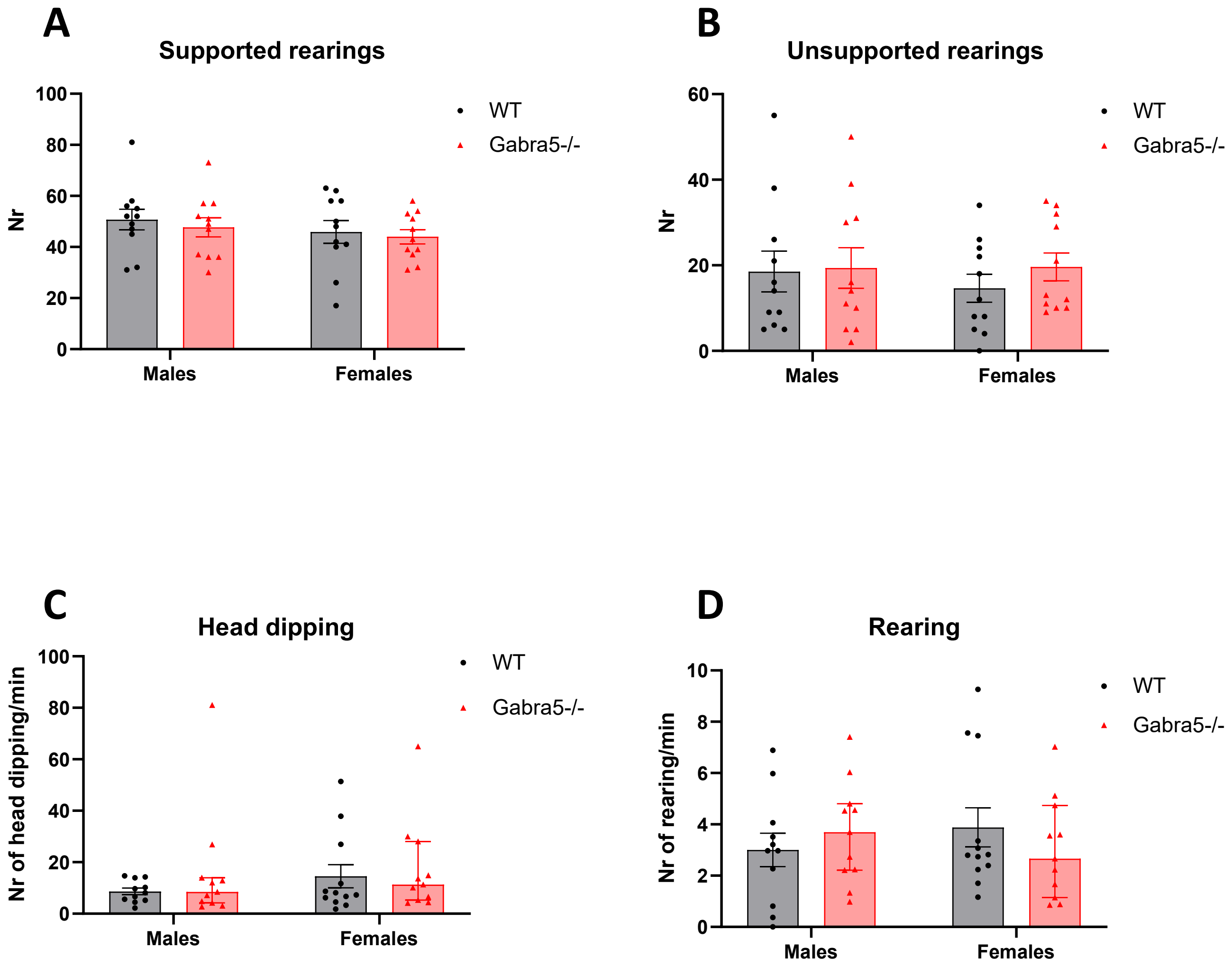

Figure S2. Rearing and head dipping. AB Number of supported and unsupported rearings in OF during 10 min, Two-way ANOVA main genotype effect p= 0.59 and p= 0.39, respectively, genotype/sex interaction p> 0.05, depicted with mean ± SEM, n=12. CD The number of head dippings and rearings normalized to time spent in the open arms of EPM. No significant differences are seen between the genotypes. Robust ANOVA main genotype effect p= 0.47 and p= 0.95, respectively, genotype/sex interaction p>0.05, depicted with median with interquartile range, n=12
